# Fourier ptychographic reconstruction using Poisson maximum likelihood and truncated Wirtinger gradient

**DOI:** 10.1038/srep27384

**Published:** 2016-06-10

**Authors:** Liheng Bian, Jinli Suo, Jaebum Chung, Xiaoze Ou, Changhuei Yang, Feng Chen, Qionghai Dai

**Affiliations:** 1 Department of Automation, Tsinghua University, Beijing 100084, China; 2 Department of Electrical Engineering, California Institute of Technology, Pasadena, CA 91125, USA

## Abstract

Fourier ptychographic microscopy (FPM) is a novel computational coherent imaging technique for high space-bandwidth product imaging. Mathematically, Fourier ptychographic (FP) reconstruction can be implemented as a phase retrieval optimization process, in which we only obtain low resolution intensity images corresponding to the sub-bands of the sample’s high resolution (HR) spatial spectrum, and aim to retrieve the complex HR spectrum. In real setups, the measurements always suffer from various degenerations such as Gaussian noise, Poisson noise, speckle noise and pupil location error, which would largely degrade the reconstruction. To efficiently address these degenerations, we propose a novel FP reconstruction method under a gradient descent optimization framework in this paper. The technique utilizes Poisson maximum likelihood for better signal modeling, and truncated Wirtinger gradient for effective error removal. Results on both simulated data and real data captured using our laser-illuminated FPM setup show that the proposed method outperforms other state-of-the-art algorithms. Also, we have released our source code for non-commercial use.

Fourier ptychographic microscopy (FPM) is a novel computational coherent imaging technique for high space-bandwidth product (SBP) imaging^1^,^2^. This technique sequentially illuminates the sample with different incident angles, and correspondingly captures a set of low-resolution (LR) images of the sample. Assuming that the incident light is a plane wave and the imaging system is a low-pass filter, the LR images captured under different incident angles correspond to different spatial spectrum bands of the sample, as shown in Fig. 1. By stitching these spectrum bands together in Fourier space, a large field-of-view (FOV) and high resolution (HR) image of the sample can be obtained. As a reference, the synthetic numerical aperture (NA) of the FPM setup reported in ref. 1 is ~0.5, and the FOV can reach ~120 mm^2^, which greatly improves the throughput of the existing microscope. FPM has been widely applied in 3D imaging^3^,^4^, fluorescence imaging^5^,^6^, mobile microscope^7^,^8^, and high-speed *in vitro* imaging^9^.

Mathematically, Fourier ptychographic (FP) reconstruction can be implemented as a typical phase retrieval optimization process, which needs to recover a complex function given the intensity measurements of its linear transforms. Specifically, we only obtain the LR intensity images corresponding to the sub-bands of the sample’s HR spatial spectrum, and aim to retrieve the complex HR spatial spectrum. Conventional FPM^1^,^2^ utilizes the alternating projection (AP) algorithm^10^,^11^, which adds constraints alternately in spatial space (captured intensity images) and Fourier space (pupil function), to stitch the LR sub-spectra together. AP is easy to implement and fast to converge, but is sensitive to measurement noise and system errors arising from numerous factors such as low signal-to-noise ratio due to short camera exposure time^12^ and incorrect sub-sampling of the HR spatial spectrum due to misalignments of the imaging system. To tackle measurement noise, Bian *et al*.^13^ proposed a novel method termed Wirtinger flow optimization for Fourier Ptychography (WFP), which uses the gradient descent scheme and Wirtinger calculus^14^ to minimize the intensity errors between estimated LR images and corresponding measurements. WFP is robust to Gaussian noise, and can produce better reconstruction results in low-exposure imaging scenarios and thus largely decrease image acquisition time. However, it needs careful initialization since the optimization is non-convex. Based on the semidefinite programming (SDP) convex optimization for phase retrieval^15^,^16^, Horstmeyer *et al*. modeled FP reconstruction as a convex optimization problem^17^. The method guarantees a global optimum, but converges slow which makes it impractical in real applications. Recently, Yeh *et al*.^18^ tested different objective functions (intensity based, amplitude based and Poisson maximum likelihood) under the gradient-descent optimization scheme for FP reconstruction. The results show that the amplitude-based and Poisson maximum-likelihood objective functions produce better results than the intensity-based objective function. To address the LED misalignment, the authors also added a simulated annealing algorithm into each iteration to search for the optimal pupil locations.

Although the above methods offer various options for FP reconstruction, they have their own limitations. AP and WFP are limited to address Gaussian noise, and cannot handle speckle noise well (as shown in following experiments) which is common when the light source is highly spatially and temporally coherent (such as laser)^12^, as well as Poisson noise and pupil location error. The Poisson Wirtinger Fourier ptychographic reconstruction (PWFP) technique mentioned in ref. 18 performs better than other methods, but it still obtains aberrant reconstruction results with the measurements corrupted with Gaussian noise, and needs much more running time for the incorporated simulated annealing algorithm to deal with LED misalignment.

In this paper, we propose a novel FP reconstruction method termed truncated Poisson Wirtinger Fourier ptychographic reconstruction (TPWFP), to efficiently handle the above mentioned measurement noise and pupil location error. The technique incorporates Poisson maximum likelihood objective function and truncated Wirtinger gradient^19^ together into a gradient-descent optimization framework. The advantages of TPWFP lie in three aspects:

- The utilized Poisson maximum-likelihood objective function is more appropriate to describe the Poisson characteristic of the photon detection by an optical sensor in real imaging systems, and thus can produce better results in real applications.
- Truncated gradient is used to prevent outliers from degrading the reconstruction, which provides better descent directions and enhanced robustness to various sources of error such as Gaussian noise and pupil location error.
- There is no matrix lifting and global searching for optimization, resulting in faster convergence and less computational requirement.

To demonstrate the effectiveness of TPWFP, we test it against the aforementioned algorithms on both simulated data and real data captured using a laser-illuminated FPM setup^12^. Both the simulations and real experiments show that TPWFP outperforms other state-of-the-art algorithms in the imaging scenarios involving Poisson noise, Gaussian noise, speckle noise and pupil location error.

## Methods

As stated before, TPWFP incorporates Poisson maximum likelihood objective function and truncated Wirtinger gradient together into a gradient descent optimization framework for FP reconstruction. Next, we begin to introduce this technique in detail.

### Image formation of FPM

**Figure 1:**
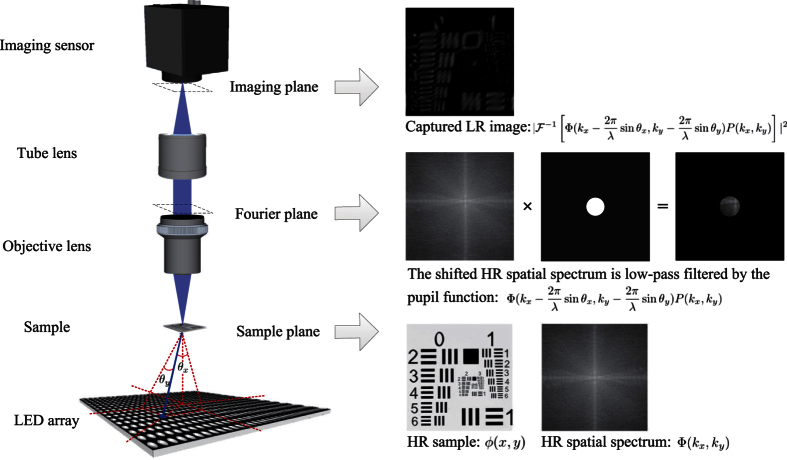
The FPM system and its image formation.

FPM is a coherent imaging system. It requires the illumination to be coherent^1^, or partially coherent^20^, in order to capture multiple images of limited spatial bandwidth information in different regions of a sample’s spatial spectrum. For a relatively thin sample^21^, different spatial spectrum regions can be accessed by angularly varying coherent illumination. Under the assumption that the light incident on a sample is a plane wave, we can describe the light field transmitted from the sample as 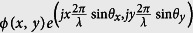, where *ϕ* is the sample’s complex spatial map, (*x, y*) are the 2D spatial coordinates, *j* is the imaginary unit, *λ* is the wavelength of illumination, and *θ*_*x*_ and *θ*_*y*_ are the incident angles as shown in Fig. 1. Then the light field is Fourier transformed to the pupil plane when it travels through the objective lens, and subsequently low-pass filtered by the aperture. This process can be represented as 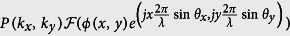, where *P*(*k*_*x*_, *k*_*y*_) is the pupil function for low-pass filtering, (*k*_*x*_, *k*_*y*_) are the 2D spatial frequency coordinates in the pupil plane, and 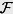 is the Fourier transform operator. Afterwards, the light field is Fourier transformed again when it passes through the tube lens to the imaging sensor. Since real imaging sensors can only capture light’s intensity, the image formation of FPM follows:


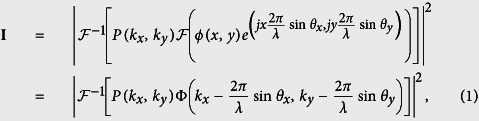


where **I** is the captured image, 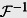 is the inverse Fourier transform operator, and Φ is the spatial spectrum of the sample. Visual explanation of the image formation process is diagrammed in Fig. 1

Because 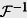 is linear and 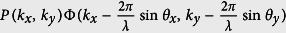 is also a linear operation that passes only a finite bandwidth of the HR spatial spectrum with the pupil function, we can rewrite the above image formation of FPM as


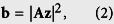


where 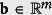 is the ideal captured images (all the captured **I** under different angular illumination in vector form), 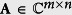 is the corresponding linear transform matrix incorporating the Fourier transform and the low-pass filtering, and 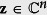 is the sample’s HR spectrum (Φ in vector form). This is a standard phase retrieval problem^22^, where **b** and **A** are known, and **z** is what we aim to recover.

### Poisson maximum-likelihood objective function

Here we assume that the detected photons at each detector unit follow Poisson distribution in real setups, which is consistent with the independent nature of random individual photon arrivals at the imaging sensor^23^. Note that although the Poisson distribution approaches a Gaussian distribution for large photon counts according to the central limit theorem, most of the captured images in FPM are dark-field images under oblique illuminations (most dark-field pixels’ values are only 0.5–5% of the camera’s full bit-depth, see the exemplar captured image shown in Fig. 1), and are thus more consistent with Poisson distribution. The reason for this stark difference in signal strength between bright-field and dark-field images is the fact that, for natural samples such as cells, most of their energy in their spatial spectrum is concentrated at the low-frequency regions^24^. Increasing exposure time and utilizing the HDR technique^1^ could increase the signal-to-noise ratio (SNR) and accommodate the dramatic difference in the signal strength between bright-field and dark-field images, but most fast image acquisition setups such as our laser-illuminated FPM system^12^ necessitate using the same exposure level for all images. In a nutshell, the signal’s Poisson probability model can be represented as


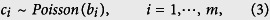


where *b*_*i*_ = |**a**_*i*_**z**|^2^ is the *i* th latent signal (pixel) in **b**, and **a**_*i*_ is the *i* th row of **A**. Thus, for *c*_*i*_, its probability mass function is


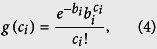


where *e* is the Euler’s number, and *c*_*i*_! is the factorial of *c*_*i*_.

Based on the maximum-likelihood estimation theory, assuming that the measurements are independent from each other, the reconstruction turns into maximizing the global probability of all the measurements *c*_1, ···, *m*_, namely


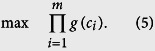


Taking a logarithm of the objective function yields


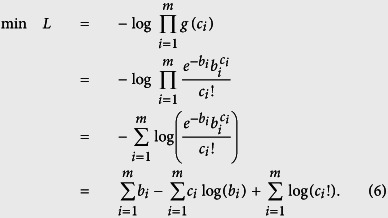


Since *c*_1, ···,*m*_ are the experimental measurements and thus constant in the optimization process, we omit the last item in *L* (namely 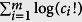) for optimization. Then by replacing *b*_*i*_ with |**a**_*i*_**z**|^2^, we obtain the objective function of TPWFP as


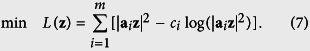


### Truncated Wirtinger gradient

As stated before, we use the gradient-descent optimization scheme. Based on the Wirtinger calculus^14^, we obtain the gradient of *L*(**z**) as


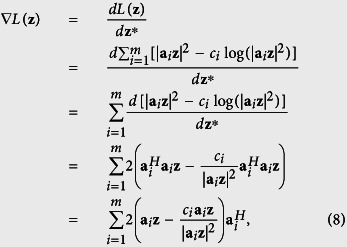


where 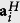 is the transposed-conjugate matrix of **a**_*i*_.

To prevent optimization degeneration from measurement noise, we add a pixel-wise thresholding operation to ∇*L*(**z**) before using it to update **z** in each iteration. Similar to ref. 19, the thresholding constraint is defined as


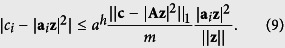


Here *a*^*h*^ is a predetermined parameter specified by users, |*c*_*i*_ − |**a**_*i*_**z**|^2^| is the difference between the *i* th measurement (pixel) and its reconstruction, 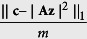 is the mean of all the differences, and 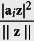 stands for the relative value of the linear transformation which is used to eliminate the scaling effect. Intuitively, the thresholding indicates that if one measurement is far from the reconstruction, it is labeled as an outlier and omitted in subsequent optimization. Note that the thresholding is signal dependent, which is beneficial for accurate detection of outliers.

Thus, for each index *i*, its corresponding measurement can be used in Eq. (8) only when it meets the thresholding criterion in Eq. (9). In the following, we use *ξ* to denote the index set that meets the thresholding constraint, and rewrite the corresponding truncated Wirtinger gradient as


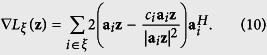


Note that the index set *ξ* is iteration-variant, meaning that in each iteration we update *ξ* according to the measurements **c** and the updated **z**. This adaptively provides us with better descent direction.

In the gradient descent scheme, we update **z** in the *k*th iteration as


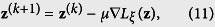


where *μ* is the gradient descent step that is predetermined manually. Here we utilize a setting similar to ref. 13 as


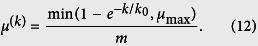


For initialization, we need to pre-determine the parameters *k*_0_ and *μ*_max_. As stated in ref. 14, the basic rule to choose their appropriate values is to make sure *μ*^(*k*)^ is small at the beginning of iterations for correct converging direction, and gradually increase it to *μ*_max_ for fast converging speed. Initiatively, *k*_0_ controls the initialization of *μ* and its increasing speed, while *μ*_max_ sets the maximum step size. Here we set *k*_0_ = 330 and *μ*_max_ = 0.1, which are experimentally validated and work well in our experiments. This type of gradient-descent step is widely used, since it allows the gradient descent step to grow gradually, and offers an adaptive way for better convergence^14^,^19^.

Aglorithm 1: TPWFP algorithm for FP reconstruction. Input: linear transform matrix **A**, measurement vector **c**, and initialization **z**^(0)^. Ouput: retrieved complex signal **z** (the sample’s HR spatial spectrum).

1. *k* = 0;
2. while not converged do
3. Update ξ according to Eq. (9);
4. Update µ^(k+1)^ according to Eq. (12);
5. Update **z**^(k+1)^ according to Eq. (11);
6. *k* := *k* + 1.
7. end

Based on the above derivations, we summarize the proposed TPWFP algorithm in Alg. 1. For the initialization **z**^(0)^, similar to ref. 13, we set **z**^(0)^ as the spatial spectrum of the up-sampled version of the LR image captured under normal incident light. According to ref. 19, the computation complexity of such an optimization algorithm is 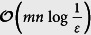, where *m* is the number of measurements, *n* is the number of signal entries, and *ε* is the relative reconstruction error defined in Eq. (13). This is much lower than WFP’s computation complexity which is 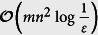. Note that the source code of TPWFP is available at http://www.sites.google.com/site/lihengbian for non-commercial use.

## Results

In this section, we test the proposed TPWFP and other three state-of-the-art algorithms including AP, WFP and PWFP on both simulated and real captured data, to show their pros and cons.

### Quantitative metric

To quantitatively evaluate the reconstruction quality, we utilize the relative error (RE)^19^ metric defined as


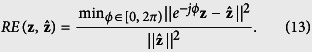


This metric describes the difference between two complex functions **z** and 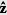. We use it here to compare the reconstructed HR spatial spectrum with its ground truth in the simulation experiments.

### Parameters

In the simulation experiments, we simulate the FPM setup with its hardware parameters as follows: the NA of the objective lens is 0.08, and corresponding pupil function is an ideal binary function (all ones inside the NA circle and all zeros outside); the height from the LED plane to the sample plane is 84.8 mm; the distance between adjacent LEDs is 4 mm, and 15 × 15 LEDs are used to provide a synthetic NA of ~0.5; the wavelength of incident light is 625 nm; and the pixel size of captured images is 0.2 um. Besides, we use the ‘Lena’ and the ‘Aerial’ image (512 × 512 pixels) from the USC-SIPI image database^25^ as the latent HR amplitude and phase map, respectively. The captured LR images’ pixel numbers are set to be one tenth of the HR image along both dimensions, and are synthesized based on the image formation in Eq. 2. We repeat 20 times for each of the following simulation experiments, and average their evaluations to produce final results.

**Figure 2:**
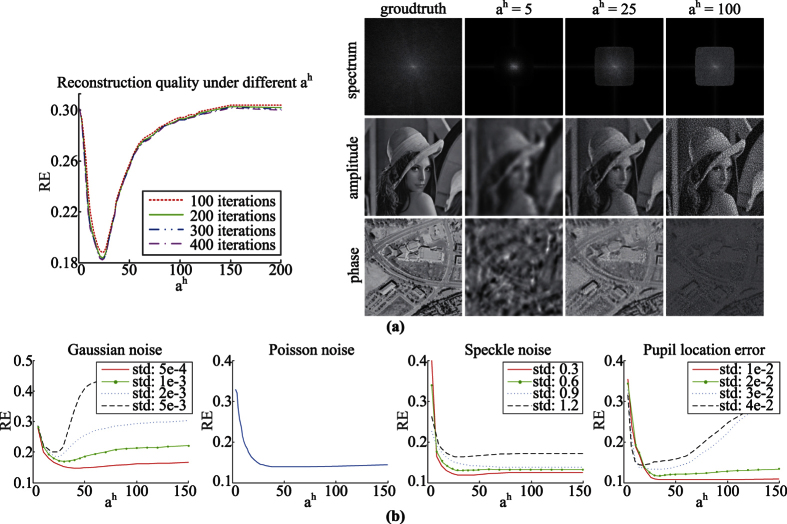
Reconstruction quality (relative error between reconstructed HR spatial spectrum and its ground truth) under different settings of *a*^*h*^ in TPWFP. (**a**) shows the reconstruction results of different iterations under Gaussian noise with the standard deviation (std) being 2e-3. (**b**) presents more results under different degeneration models (Gaussian noise, Poisson noise, speckle noise and pupil location error) and varying degeneration levels, with the iteration number being fixed at 200 which is enough for TPWFP’s convergence.

As for the algorithms’ parameters, an important parameter of TPWFP is the thresholding *a*^*h*^ in Eq. 9. To choose appropriate *a*^*h*^, we test different *a*^*h*^ on the simulated data corrupted with Gaussian noise, Poisson noise, speckle noise and pupil location error under varying degeneration levels, and study its influence on the final reconstruction quality. The results are shown in Fig. 2. Note that the standard deviation (std) is the ratio between actual std and the maximum of the ideal measurements **b**. We use the model **c** = **b**(1 + **n**) to simulate speckle noise, where **n** is uniformly distributed random noise with zero mean. Also, we simulate the pupil location error by adding Gaussian noise to the incident wave vectors of each LED. From the results we can see that both too small or too big of *a*^*h*^ result in worse reconstruction. This is determined by the nature of the utilized truncated gradient. When *a*^*h*^ is too small, more informative measurements are incorrectly labeled as outliers and thus contribute nothing to final reconstruction, resulting in less information in the recovered spatial spectra and blurred reconstructed images, as shown in Fig. 2(a). When *a*^*h*^ is too big, measurement noise and system errors are not effectively removed from the reconstructed images. To sum up, we choose an appropriate assignment for *a*^*h*^ as *a*^*h*^ = 25 in the following experiments for TPWFP, which produces satisfying results in different degeneration cases. Note that the constant assignment is reasonable because the thresholding constraint in Eq. (9) is independent from the degeneration model and level. The similar constant assignments of such parameters in ref. 19 also validate this.

Another parameter for all the algorithms is the iteration number. For different methods, we choose corresponding appropriate iteration numbers that ensure their convergence to demonstrate their best performance, but not increase unnecessary running time for fair comparison. For AP, 100 iterations are enough as proved in ref. 1. We set 1000 iterations for WFP according to ref. 13. From Fig. 2(a) we can see that 200 iterations are enough for TPWFP to converge. Since PWFP is a particular form of TPWFP when *a*^*h*^ = ∞ (no thresholding to the gradient), we set the same iteration number for PWFP.

### Simulation experiments

**Figure 3:**
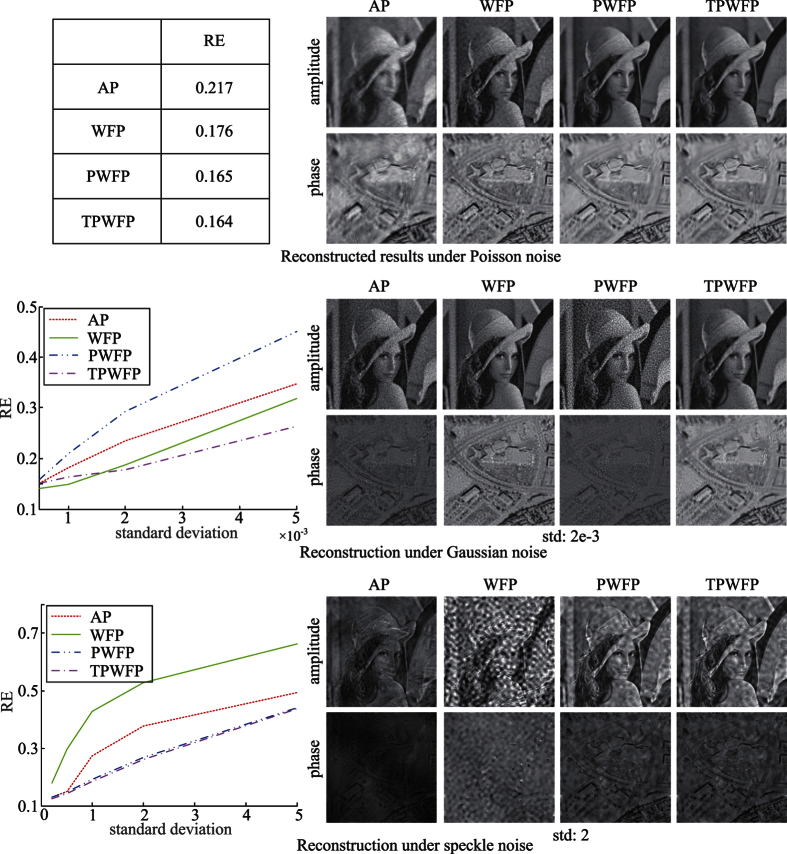
Comparison of the reconstruction results by the three state-of-the-arts (AP, WFP, PWFP) and the proposed TPWFP under Poisson noise, Gaussian noise and speckle noise.

First, we test the four algorithms (AP, WFP, PWFP and TPWFP) on simulated captured images corrupted with Poisson noise, Gaussian noise and speckle noise, respectively, which are the most common noise in real imaging setups. The first two kinds are mostly caused by photoelectric effect and dark current^23^, while speckle noise is caused by the light’s spatial-temporal coherence of the illumination source (such as laser). The results are shown in Fig. 3, from which we can see that under small Gaussian noise, WFP outperforms the other three methods. This benefits from its Gaussian noise assumption. Instead, PWFP and TPWFP only assume Poisson signal model. Thus, they cannot recognize the small Gaussian noise and remove them. However, when noise grows to around 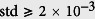, TPWFP obtains better results than WFP. This is because when noise is large, WFP cannot extract useful information from the noisy data, while TPWFP recognizes these measurements as outliers using Eq. (9) and directly omits them to avoid their negative influence on final reconstruction. For Poisson noise and speckle noise, while both PWFP and TPWFP obtain better results than the other methods, TPWFP is little advantageous than PWFP. This is because for these kinds of signal dependent noise, it is hard for the truncated gradient to correctly distinguish noise from latent signals.

**Figure 4:**
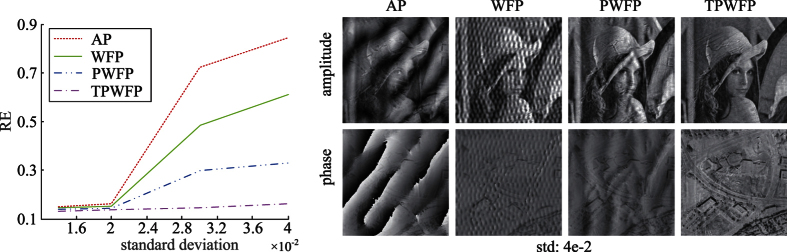
Reconstruction results by the three state-of-the-arts (AP, WFP, PWFP) and the proposed TPWFP under pupil location error.

Then we apply the four algorithms on the simulated data corrupted with pupil location error, which is common in real setups due to LED misalignment or unexpected system errors. The reconstruction results are shown in Fig. 4. From the results we can see that TPWFP outperforms state-of-the-arts a lot. This benefits from the nature of the utilized truncated gradient. In the thresholding operation (Eq. (9)), if one measurement (spatial space) is far from the reconstruction due to pupil location error, we omit this measurement which represents misaligned information. Thus, we prevent the pupil location error from degenerating final reconstruction.

We display the running time of each method under the current algorithm settings in Table 1. All the four algorithms are implemented using Matlab on an Intel Xeon 2.4 GHz CPU computer, with 8 G RAM and 64 bit Windows 7 system. From the table we can see that both PWFP and TPWFP save more running time than WFP due to faster convergence^19^, but they are still more time consuming than AP. Note that TPWFP consumes more time than PWFP, which is caused by the additional thresholding and truncation operation to the gradient.

### Real experiment

**Figure 5:**
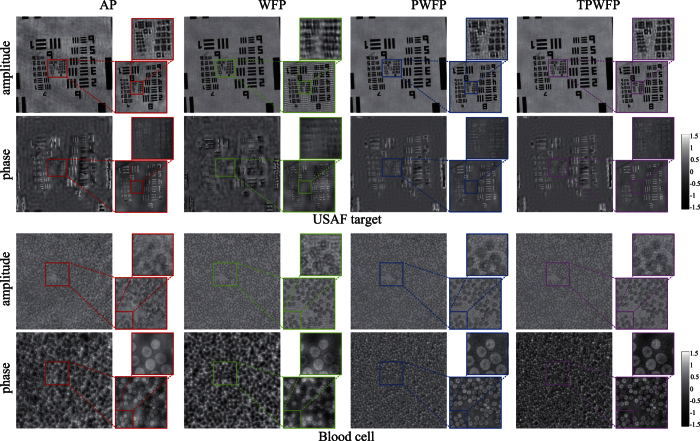
Reconstruction results by the three state-of-the-arts (AP, WFP, PWFP) and the proposed TPWFP under real captured dataset (USAF target and red blood cell) using our laser-illuminated FPM setup.

To further validate the robustness of TPWFP to the above measurement noise and system errors, we run the four algorithms on two real captured datasets including USAF target and red blood cell sample using a laser-illuminated FPM setup^12^. The red blood cell sample is prepared on a microscope slide stained with Hema 3 stain set (Wright-Giemsa). The setup consists of a 4*f* microscope system with a 4 × 0.1 NA (corresponding pupil function is still assumed to be known as an ideal binary function, same as the simulation experiments) objective lens (Olympus), a 200 mm focal-length tube lens (Thorlabs), and a 16-bit sCMOS camera (PCO.edge 5.5). The system is fitted with a circular array of 95 mirror elements providing illumination NA of 0.325, resulting in the total synthetic NA of 0.425. A 1W laser of 457 nm wavelength is used for the illumination source, which is pinhole-filtered, collimated and guided to a pair of Galvo mirrors (Thorlabs GVS212) to be directed to individual mirror elements. The use of a high-power laser allows for fast total capturing time at 0.96 seconds, but the high coherence of the laser source introduces speckle artifacts originating from reflective surfaces along the optical path, manifesting themselves as slowly varying fringe patterns. The reconstruction results are shown in Fig. 5. From the results we can see that AP produces intensity fluctuations in the background (see the white background of the USAF target for clear comparison) and low image contrast (see the reconstructed amplitude of the red blood cell sample). WFP also obtains corrugated artifacts due to the speckle noise produced by the laser illumination. Both PWFP and TPWFP obtain better results than AP and WFP, while TPWFP produces results with more image details (see the reconstructed amplitude of the USAF target, especially group 10) and higher image contrast (see the reconstructed phase of the red blood cell sample) than PWFP. To conclude, TPWFP outperforms the other methods with less artifacts, higher image contrast and more image details.

## Discussion

In this paper, we propose a novel reconstruction method for FPM termed as TPWFP, which utilizes Poisson maximum-likelihood objective function and truncated Wirtinger gradient for optimization under a gradient descent framework. Results on both simulated data and real data captured using our laser-illuminated FPM setup show that the proposed method outperforms other state-of-the-art algorithms in the cases of Poisson noise, Gaussian noise, speckle noise and pupil location error.

We note that the proposed TPWFP does not assume Poisson noise model and only target Poisson noise. Instead, it is the process of the photons arriving at the detector that is being assumed to be Poisson-distributed. The final measurements by the sensor may be corrupted with various noise such as thermal noise in the detector and pupil location error. Also, though both TPWFP and PWFP use the Poisson signal model, the difference between them is the additional gradient truncation of TPWFP. Thus, if the signal is corrupted with noise (whatever noise type), the additional gradient truncation procedure in TPWFP can remove outliers and produce noise-free reconstruction. On the contrary, for PWFP with no noise removal procedure, the noise in the measurements would propagate to the final reconstruction and largely degenerate the algorithm’s performance.

TPWFP can be widely extended. First, the pupil function updating procedure of the EPRY-FPM algorithm^26^ can be incorporated into TPWFP to obtain corrected pupil function and better reconstruction. Second, other more robust and faster optimization schemes such as the conjugate gradient method^27^ can be applied to TPWFP to further improve its performance. Third, since the linear transform matrix **A** can be composed of any kinds of linear operations (Fourier transform and low-pass filtering in FPM), TPWFP can be applied in various linear optical imaging systems for phase retrieval, such as conventional ptychography^28^, multiplexed FP^20^,^29^ and fluorescence FP^5^. Fourth, since TPWFP is much more robust to pupil location error than other methods, it may find wide applications in other imaging schemes where precise calibrations are unavailable.

In spite of the advantageous performance and wide applications, the limitations of TPWFP lie in two aspects. First, it is still time consuming compared to conventional AP, though it is much faster than WFP. Second, it is non-convex. Although choosing the up-sampled image captured under normal illumination as the initialization results in satisfying reconstruction as we demonstrate in the above experiments, there is no theoretical guarantee for its global optimal convergence.

## Additional Information

**How to cite this article**: Bian, L. *et al*. Fourier ptychographic reconstruction using Poisson maximum likelihood and truncated Wirtinger gradient. *Sci. Rep.* **6**, 27384; doi: 10.1038/srep27384 (2016).

## Figures and Tables

**Table 1:** Comparison of running time between state-of-the-arts and the proposed TPWFP.

	AP	WFP	PWFP	TPWFP
Iteration	100	1000	200	200
Running time (s)	37	301	65	117
